# Research on Fault Extraction Method of Variational Mode Decomposition Based on Immunized Fruit Fly Optimization Algorithm

**DOI:** 10.3390/e21040400

**Published:** 2019-04-15

**Authors:** Jie Zhou, Xiaoming Guo, Zhijian Wang, Wenhua Du, Junyuan Wang, Xiaofeng Han, Jingtai Wang, Gaofeng He, Huihui He, Huiling Xue, Yanfei Kou

**Affiliations:** School of Mechanical Engineering, North University of China, Taiyuan 030051, China

**Keywords:** VMD, IFOA, permutation entropy, fault diagnosis

## Abstract

In recent years, a new method of fault diagnosis, named variational mode decomposition (VMD), has been widely used in industrial production, but the decomposition accuracy of VMD is determined by two parameters, which are respectively the decomposition layer number k and the penalty factor α, if the parameters are not properly selected, there will be over-decomposition or under-decomposition. In order to find an approach to determine the parameters adaptively, a method to optimize VMD by using the immune fruit fly optimization algorithm (IFOA) is proposed in this paper. In this method, permutation entropy is used as the fitness function, firstly, the immune fruit fly optimization algorithm is used to search the combined parameters of k and α in VMD, searching for the best combination parameters of k and α by iteration, and then uses the combined parameters to perform VMD, finally, the center frequency is determined through frequency spectrum analysis. The method mentioned is applied to the fault extraction of a simulated signal and a measured signal of a wind turbine gearbox, and the fault frequency is successfully extracted. Using ensemble empirical mode decomposition (EEMD) and singular spectrum decomposition (SSD) to compare with the proposed method, which validated feasibility of the proposed method.

## 1. Introduction

As an important part of rotating machinery, gearboxes are widely used in industrial fields [1,2,3]. Due to the harsh working environment of the gearbox, the fault of the gearbox is inevitable. It is useful to diagnose the type of fault and the fault location before gearbox out of action. So, composite fault extraction of the gearbox is critical [4,5,6]. At present, the methods used in gearbox fault extraction include Wavelet transform (WT), Empirical mode decomposition (EMD), Local mean decomposition (LMD), and Ensemble empirical mode decomposition (EEMD), these methods successfully extract the fault information, but they will show their own weaknesses when extracting composite faults [7,8,9,10]. When using WT to decompose signals, we need to give a basis function and the number of decomposing layers ahead of time, that is, WT is not adaptive [11,12,13,14,15]. The flaw of EMD is that its results show the phenomenon of mode aliasing and endpoint effects. The decomposition result of LMD is greatly affected by the step size [16,17,18,19,20]. EEMD is developed on the basis of EMD, it is adaptively decomposed by adding white noise to the original signal and calculate mean of the intrinsic mode function (IMF), but the amplitude of the added white noise has a great influence on its decomposition result. In order to achieve the desired extraction effect, many scholars have improved these methods, but most of them are denoising, which cannot solve the original problem fundamentally [21,22,23,24,25]. In 2014, Dragomiretskiy and Zosso proposed variational mode decomposition (VMD), and it was first applied in the field of communication. With the research of VMD in many scholars, it is was then applied in industry and other fields [26].

Jiang et al. [27] found U-shaped convergence phenomenon when studying initial center frequencies (ICFs) and proposed a VMD method using the energy fluctuation spectrum to find the initial center frequency and extract fault information. Wang et al. [28] qualitatively analyzed the high efficiency and high robustness of VMD by comparing VMD with empirical wavelet transform (EWT), EMD, and EEMD. Shen et al. [29] used VMD to denoise micro-electro-mechanical system gyroscopes and obtained the ideal denoising result through experiments. Jiang et al. [30] used adaptive multiresonance bands detection based on VMD and using Multiresolution Teager Energy Operator (MTEO) to enhance the rolling element bearing the fault diagnosis. With the deepening of research, the advantages of VMD gradually manifest, but it still has the problem that parameters k and α often depended on human selection. In order to solve such problems, Yan et al. [31] used the cross-correlation criterion to determine the decomposition layer k of VMD adaptively, but ignored the influence of the penalty factor. Liao et al. [32] used the correlation coefficient to determine the value of the penalty factor α and used the optimized VMD in the magnetocardiogram (MCG) to verify the effectiveness of the proposed method, but it did not discuss influence of the decomposition layer k. Miao et al. [33] used ensemble kurtosis to improve the grasshopper optimization algorithm, and then select k adaptively, but the proposed method could not guarantee the global optimality.

The fruit fly optimization algorithm put forward in 2011. As a group optimization algorithm, it is similar to the particle swarm optimization algorithm (PSO), artificial fish swarm optimization algorithm, and firefly optimization algorithm. Fruit fly optimization algorithm has been used in many fields because of its simple theory that is easy to understand and short computation time [34]. Wang et al. [35] optimized the parameters of the least squares support vector machine (LSSVM) with the improved fruit fly optimization algorithm, the optimized results were compared with those of PSO optimization, and the advantages of fruit fly optimization algorithm were quantitatively analyzed through four indicators. Wang et al. [36] took level probability policy (LP) as the objective function of fruit fly optimization algorithm, and successfully solved the optimization problem of twenty-nine continuous complex functions. Sheng et al. [37] optimized the PID controller by using the fruit fly optimization algorithm to optimize the three parameters of the PID. Li et al. [38] used the fruit fly optimization algorithm to solve the semiconductor final testing scheduling problem.

Artificial immune algorithm is an intelligent algorithm that simulates the biological immune system, including cloning, mutation, antigen input and antibody generation, etc. Due to its high computational accuracy, this algorithm has been used in many fields after improvement and research by many researchers [39,40,41]. By expanding the immune system, Liu et al. [42] took the leakage area of the steam belt in the tire workshop as an antigen and realized the location of its leakage. Tvana et al. [43] used immune algorithms to classify computer products and put it in production practice. Fernand p. Lima et al. [44] completed the fault diagnosis of the power system by using the immune algorithm to monitor the protection system of distribution network. Gong et al. [45] applied the artificial immune algorithm to medical image processing, and the enhancement of magnetic resonance image was realized by filtering part of the noise.

## 2. Basic Theory

**Figure 1:**
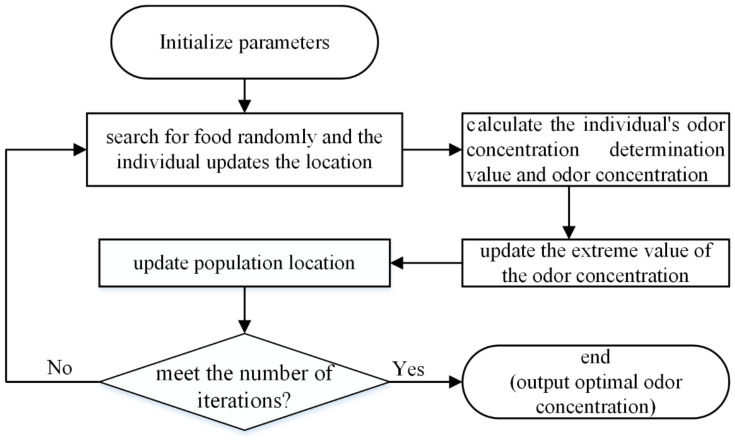
Fruit fly optimization algorithm.

Fruit fly optimization algorithm (FOA) is an intelligent algorithm proposed in 2011 that simulates the group foraging behavior of fruit flies. It takes the optimization target as the food source, it quickly finds the optimization target through powerful olfactory search and visual positioning. The steps are as follows:

(1) Initialize parameters: number of fruit flies, number of iterations, and initial position of the population.
(2) According to its sense of smell, fruit flies randomly search for food and arrive at a new location.
(3) Calculate the odor concentration determination value of the individual.
(4) Calculate the odor concentration of the individual where the fruit fly is located.
(5) Find the minimum of odor concentrations.
(6) Record the location and the odor concentration value of the fruit fly in step (5). Other fruit flies fly to the location when the visual positioning function is open.
(7) Iterative operation, repeating steps (2) to (6). In the iteration, if the minimum odor concentration value is less than that of the previous generation, the optimal solution is updated until the number of iterations reaches the set value. The flow chart of the proposed method is shown in Figure 1.

**Figure 2:**
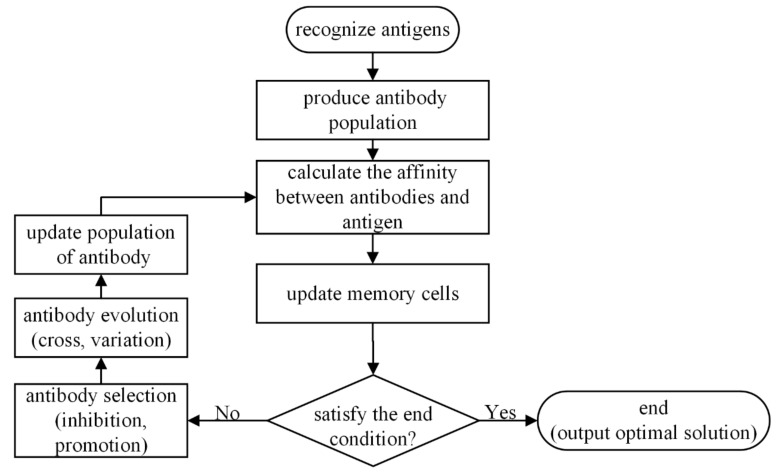
Artificial immune algorithm.

In the artificial immune algorithm, the antigen is the problem and the antibody is the solution to the problem. The best antibody obtained is the optimal solution of the objective function, which updates the population of the antibody through the steps of mutation, selection, and inhibition. The process is shown in Figure 2.

### 2.1. Immune Fruit Fly Optimization Algorithm

IFOA combines the fruit fly optimization algorithm with the artificial immune algorithm. Adding the variation and vaccination steps of the artificial immune algorithm to the fruit fly optimization algorithm. The steps are:

(1) Set parameters: Given the number of fruit flies Swarmsize, the maximum number of iterations MaxIter, the dimension of optimization Dim, and the initial position of the population $\left(X_{axis},Y_{axis}\right)$, the initial position is obtained randomly.

(2) Give the flight direction and distance of the individual fruit fly randomly and reach the new position $\left(X_{i},Y_{i}\right)$. Since the position of the food is unknown, the distance of fruit fly to the origin needs to be calculated. Then, calculating the odor concentration determination values of the individual $S_{i}$ its smell concentration determination value $S_{i}$ is the reciprocal of the distance between the location and the origin, $random_{i}^{1},random_{i}^{2}$$random_{i}^{1},random_{i}^{2}$ are the movement steps in the X and Y directions, respectively.

(1) $$\left.\begin{array}{c} X_{i}=X_{axis}+rangdom_{i}^{1}\\ Y_{i}=Y_{axis}+rangdom_{i}^{2} \end{array}\right\}.$$

(2) $$S_{i}=\frac{1}{\sqrt{X_{i}^{2}+Y_{i}^{2}}}.$$

(3) Calculate the corresponding odor concentration $Smell_{i}$ of individuals through the calculated $S_{i}$ in step (2).

(3) $$Smell_{i}=Fuction(S_{i}).$$

(4) Find the optimal odor concentration.

(4) $$\left[bestSmell,bestindex\right]=\min\left(Smell_{i}\right).$$

(5) The fruit fly corresponding to the optimal odor concentration was taken as the immune vaccine, and the group flew to the individual.

(5) Smellbest=bestSmell

(6) $$\left.\begin{array}{c} X_{axis}=X\left(bestSmell\right)\\ Y_{axis}=Y\left(bestSmell\right) \end{array}\right\}.$$

(6) In the way of roulette, the probability of $P_{i}$ was used to perform mutation operation on M fruit flies. The higher the odor concentration value of individual was, the higher the probability of mutation was. Whenever a random number is generated between [0,1], the location of the mutant fruit fly is updated according to formula (9), where λ = 10, the location of the fruit fly before the mutation is $\left(X_{j},Y_{j}\right)$, and the location of the individual after the mutation is $\left(X_{j}^{\prime },Y_{j}^{\prime }\right)$,j=1,2…M.

(7) $$f_{i}=\frac{1}{Smell_{i}}.$$

(8) $$P_{i}=\frac{f_{i}}{\sum_{i=1}^{Swarmsize}f_{i}}.$$

(9) $$\left.\begin{array}{c} X_{j}^{\prime }=X_{j}+\lambda \times X_{axis}\\ Y_{j}^{\prime }=Y_{j}+\lambda \times Y_{axis} \end{array}\right\}.$$

(7) Calculate the odor concentration determination value $S_{j}^{\prime }$ and the corresponding odor concentration value $Smell_{j}^{\prime }$ of the mutant fruit fly, and update the odor concentration extremum.

(8) After the mutant fruit flies were added to the population, formula (10) was used to select Swarmsize fruit flies to form a new population. K fruit flies were inoculated with the immune vaccine generated in step (5), and the immune selection was conducted according to the odor concentration value of individual fruit flies before and after vaccination. If the odor concentration value decreases, the corresponding individual will be retained; otherwise, the vaccine will be cancelled.

(10) $$P_{k}=\frac{\sum_{i,k=0}^{Swarmsize+M}\left|f\left(x_{i}\right)-f\left(x_{k}\right)\right|}{\sum_{i=0}^{Swarmsize+M}\sum_{k=0}^{Swarmsize+M}\left|f\left(x_{i}\right)-f\left(x_{k}\right)\right|}.$$

(9) Iterate from step (2) to step (8) to update the optimal solution until the maximum number of iterations.

### 2.2. VMD Algorithm

VMD is a new decomposition method with a solid mathematical foundation. Firstly, the corresponding constrained variational model expression is established, then, the penalty factor and the Lagrange function are introduced to change the constrained variational problem into unconstrained variational problem. Finally, the center frequency and bandwidth of each decomposition mode function are found by iteratively solving this problem. The result is that IMFs are obtained and its specific process is:

(1) Input signal $x\left(t\right)$ and perform a Hilbert transform on each modal function:$u_{k}\left(t\right)$.

(11) $$\left(\delta \left(t\right)+\frac{j}{\pi t}\right)*u_{k}\left(t\right)$$

In the formula (11),t is time,$\delta \left(t\right)$is the impact function, ∗is the convolution operation, and $\left\{u_{k}\left(t\right)\right\}=\left\{u_{1}\left(t\right),u_{2}\left(t\right)\ldots ,u_{k}\left(t\right)\right\}$ is the decomposed IMF.

(2) Combing the center frequency $\omega_{k}$ with the obtained analytical signal, and shifting the frequency spectrum to baseband:

(12) $$\left[\left(\delta \left(t\right)+\frac{j}{\pi t}\right)*u_{k}\left(t\right)\right]e^{-j\omega_{k}t}$$

where $\left\{\omega_{k}\right\}=\left\{\omega_{1},\omega_{2}\ldots ,\omega_{k}\right\}$ is the center frequency of the corresponding IMF.

(3) Gaussian smoothing is used to estimate the bandwidth $u_{k}\left(t\right)$ of after frequency shifting. Construct a constrained variational model. Whose expression is:

(13) $$\left.\begin{array}{c} \underset{\left(u_{k})(\omega_{k}\right)}{\min}\left\{\sum_{k}\left\|{\left.\partial_{t}\left[\left(\delta (t)+\frac{j}{\pi t})*u_{k}(t\right)\right]e^{-j\omega_{k}t}\right\|}_{2}^{2}\right.\right\}\\ s.t.\sum_{k}u_{k}=x(t) \end{array}\right\}$$

where, $\partial_{t}$ is the partial derivative with respect to time, $s.t.\sum_{k}u_{k}=u_{1}+u_{2}+\ldots +u_{k}$. 

(4) Penalty factor α and Lagrange multiplication operator $\lambda \left(t\right)$ are introduced to construct the unconstrained variational problem. The mathematical expression of the augmented Lagrange function is as follows:

(14) $$L(\left\{u_{k}\right\},\left\{\omega_{k}\right\},\lambda )=\alpha {\left.\sum_{k}\left\|\left[(\sigma (t)+\frac{j}{\pi t}\right)\right.*u_{k}(t)]e^{-j\omega_{k}t}\right\|}_{2}^{2}+{\left.\left\|x(t)-\sum_{k}u_{k}(t)\right.\right\|}_{2}^{2}+\langle \lambda (t),x(t)-\sum_{k}u_{k}(t)\rangle$$

(5) Perform frequency domain conversion on step (4). In order to obtain an optimal solution of the constrained variational model, the alternate direction method of multipliers (ADMM) is used to calculate the saddle point of the augmented Lagrange function. The saddle-point problem is solved by the alternate renewal of components $u_{k}^{n+1},\omega_{k}^{n+1},\lambda^{n+1}$, the expression of updating $u_{k}^{n+1},\omega_{k}^{n+1},\lambda^{n+1}$ is

(15) $${\overset{\wedge }{u}}_{k}^{n+1}(\omega )=\frac{\overset{\wedge }{f}\left(\omega \right)-\sum_{i\neq k}{\overset{\wedge }{u}}_{i}(\omega )+\frac{\overset{\wedge }{\lambda }(\omega )}{2}}{1+2\alpha {\left(\omega -\omega_{k}\right)}^{2}}.$$

(16) $$\omega_{k}^{n+1}=\frac{{\int_{0}^{\infty }\omega \left|{\overset{\wedge }{u}}_{k}(\omega )\right|}^{2}d\omega }{{\int_{0}^{\infty }\left|{\overset{\wedge }{u}}_{k}(\omega )\right|}^{2}d\omega }.$$

(17) $${\overset{\wedge }{\lambda }}^{n+1}(\omega )={\overset{\wedge }{\lambda }}^{n}(\omega )+\tau (\overset{\wedge }{f}(\omega )-\sum_{k}{\overset{\wedge }{u}}_{k}^{n+1}(\omega )).$$

The algorithm steps are:

(1) Initialize parameters: Set $\left\{{\overset{\wedge }{u}}_{k}^{1}\right\}$, $\left\{{\overset{\wedge }{\omega }}_{k}^{1}\right\}$, ${\overset{\wedge }{\lambda }}^{1}$, n to 0.

(2) Update ${\overset{\wedge }{u}}_{k}^{n+1}(\omega )$, $\omega_{k}^{n+1}$ and ${\overset{\wedge }{\lambda }}^{n+1}(\omega )$ respectively by using the formulas (14)–(16).

(3) Iterative step (2) until the termination condition is satisfied $\frac{\sum {}_{k}{\left\|{\overset{\wedge }{u}}_{k}^{n+1}-{\overset{\wedge }{u}}_{k}^{n}\right\|}_{2}^{2}}{\left\|{\overset{\wedge }{u}}_{k}^{n}\right\|}<\varepsilon$, and finally k IMFs are obtained. Where ε is tolerance parameter of the convergence criterion that usually in the value of 10^−7^.

### 2.3. VMD Based on the Immune Fruit Fly Optimization Algorithm

Fruit fly optimization algorithm has the characteristics of small calculation and fast convergence, but it is easy to fall into a local optimal solution. On the contrary, the artificial immune algorithm has a large amount of calculation and low efficiency, but its calculation accuracy is high. Combining the artificial immune algorithm with the fruit fly optimization algorithm, the fitness of the artificial immune algorithm is calculated by the odor concentration of the fruit fly algorithm. The fruit fly individual corresponds to the antibody. The optimal individual in the fruit fly optimization algorithm is used as the immune vaccine, and the mutation and immune selections of the artificial immune algorithm are added to fruit fly optimization algorithm. Adding variation and immune selection of artificial immune algorithm into the fruit fly optimization algorithm, it can increase the diversity of the fruit fly population and effectively avoid the problem that the fruit fly optimization algorithm falls into the local optimal solution. VMD can only decompose one mode component at a time, and there is no mode aliasing problem. The decomposition accuracy is high, but the decomposition layer number k and penalty factor α need to be given according to experience, and the choice of k and α is very important for fault extraction. Therefore, this paper adopts the immune fruit fly optimization algorithm to simultaneously optimize the k and α of VMD, and automatically search for the best combination of k and α. When using the immune fruit fly optimization algorithm to optimize the parameters of the VMD, it is necessary to select a fitness function to continuously update the position of the fruit fly population. Permutation entropy (PE) is a parameter for measuring the complexity of a time series. The IMF, after the decomposition of the original signal, is processed into a time-distributed sequence, and the calculated entropy value reflects the complexity of the signal. The principle of permutation entropy is as follows:

It is assumed that a one-dimensional time series $\left\{x(i),i=1\sim N\right\}$ with a length of *N* is assumed. Set the embedding dimension to m and the delay time to τ (the value of τ is usually 1), and performs phase space reconstruction to obtain a matrix of the following form:

(18) $$\left[\begin{array}{cccc} x(1) & x(1+\tau ) & \cdot \cdot \cdot & x(1+(m-1)\tau )\\ \cdot \cdot \cdot & \cdot \cdot \cdot & \cdot \cdot \cdot & \cdot \cdot \cdot \\ x(j) & x(j+\tau ) & \cdot \cdot \cdot & x(j+(m-1)\tau )\\ \cdot \cdot \cdot & \cdot \cdot \cdot & \cdot \cdot \cdot & \cdot \cdot \cdot \\ x(K) & x(K+\tau ) & \cdot \cdot \cdot & x(K+(m-1)\tau ) \end{array}\right]$$

wherej=1,2,…K,Kis the number of rows of the matrix, that is, the number of reconstructed vectors, K=N−(m−1)τ.

Arrange the m reconstruction matrices of each row from large to small:

(19) $$x(i+(j_{1}-1)\tau )\leq x(i+(j_{2}-1)\tau )\leq \cdot \cdot \cdot \leq x(i+(j_{m}-1)\tau ).$$

Assuming that there are equal elements in the reconstructed vector, the two elements are arranged in the original order. Therefore, we can obtain a corresponding symbol sequence$S(l)=(j_{1},j_{2},\ldots ,j_{m})$ for any reconstituted vector $x\left(j\right)$, where l=1,2,…k, k≤m!.

For the symbol sequence corresponding to the k reconstruction vectors of the one-dimensional time series x(i). The expression of its permutation entropy is:

(20) $$PE_{P}(m)=-\sum_{j=1}^{k}P_{j}\ln P_{j}$$

where $P_{j}$ is the probability of occurrence of any time series [46].

When a composite signal is decomposed by VMD, if the noise content in the IMF is less, the permutation entropy of the IMF is smaller. Optimize the decomposition layer number k and the penalty factor α in VMD by using the immune fruit fly optimization algorithm. Each fruit fly is a possible solution. A set of (k, α) corresponding to the location of a fruit fly is input into VMD for decomposition. Then, calculating the permutation entropy of the decomposed IMFs and the minimum value is taken as the optimal odor concentration. The steps to optimize k and α are:

(1) Initialize parameters: population size Swarmsize, maximum number of iterations MaxIter, optimization dimension Dim, and initial position of the population $\left(X_{axis},Y_{axis}\right)$. $X_{axis}$ and $Y_{axis}$ are a matrix of one row and two columns, that is to say, the optimization dimension Dim=2.

(2) According to formula (1), giving the flight step size of each fruit fly randomly $random_{i}^{1},random_{i}^{2}$.

(3) In the formula (2), $S_{i}$ contains $S_{i,1}$ and $S_{i,2}$. $S_{i,1}$ corresponds to decomposition layer, $S_{i,2}$ corresponds to penalty factor. Formula (3) can be written as:

(21) $$Smell_{i}=Fuction\left(k,\alpha \right)=PE(IMFs).$$

Using (k, α) corresponding to each fruit fly position to perform VMD and calculating the permutation entropy of the decomposed IMFs.

**Figure 3:**
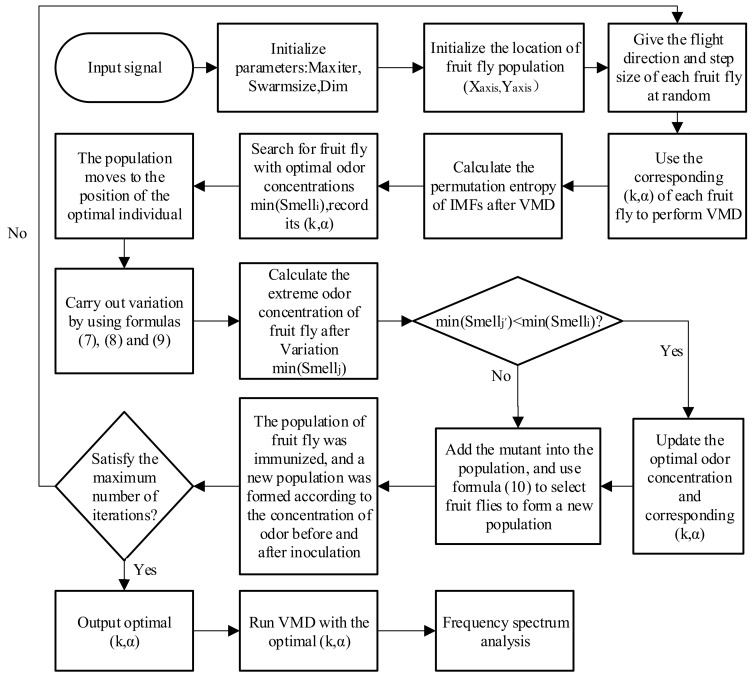
Flow chart of the proposed method. (intrinsic mode function (IMF); variational mode decomposition (VMD)).

(4) Search for the fruit fly have the optimal odor concentration value through formula (4), which means looking for individual with the smallest permutation entropy, and record values of k and α.

(5) According to formula (5) and formula (6), start visual search and update the location of the population.

(6) Calculate mutation and vaccination according to formulas (7)–(10). Calculate the minimum permutation entropy in the population after variation and inoculation, and record the corresponding k and α.

(7) Search for optimal fruit fly individual by the iterative method and record its k and α.

(8) Input the optimal combination of k and α into the VMD and then analyze its frequency spectrum. Its specific flow chart is shown in Figure 3.

## 3. Simulation Analysis

### 3.1. Effects of k and α on VMD

To illustrate the effect of k and α on the VMD results, a simulated signal, as shown in formula (19), is constructed:

(22) $$\left\{\begin{array}{l} x_{1}\left(t\right)=2\sin\left(2\pi f_{1}t\right)\\ x_{2}\left(t\right)=1.5\sin\;\left(2\pi f_{2}t\right)\\ x_{3}\left(t\right)=1\sin\;\left(2\pi f_{3}t\right)\\ x\left(t\right)=x_{1}\left(t\right)+x_{2}\left(t\right)+x_{3}\left(t\right) \end{array}\right..$$

**Figure 4:**
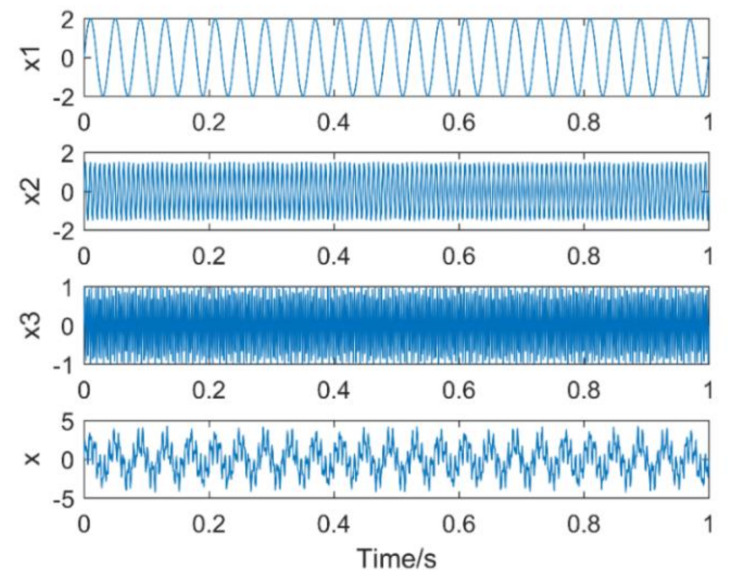
Time domain diagram of each component signal and simulation signal.

**Figure 5:**
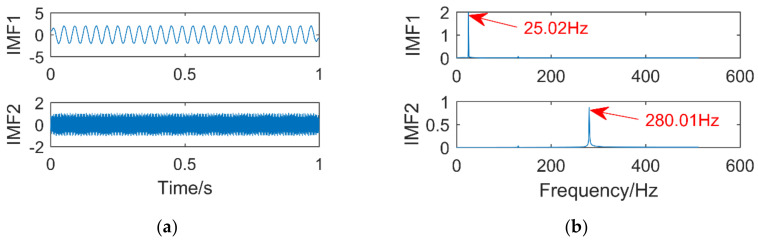
Time domain diagram (**a**) and Frequency domain diagram(**b**) of VMD.

**Figure 6:**
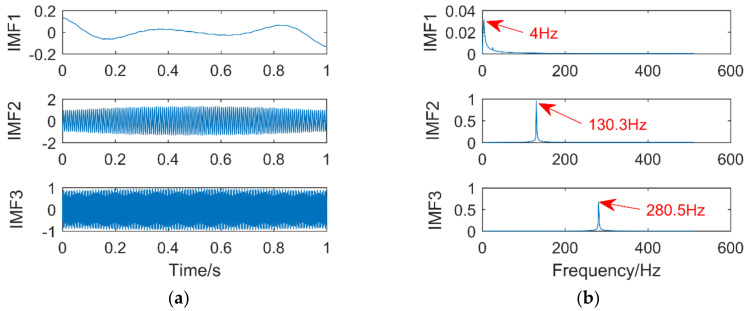
Time domain diagram (**a**) and Frequency domain diagram(**b**) of VMD.

The simulation signal consists of three sinusoidal signals,$f_{1}$ = 25 Hz,$f_{2}$ = 130 Hz,$f_{3}$ = 280 Hz. The sampling points are 1024, and the sampling frequency is 1024 Hz. The time-domain diagram of the simulation signal is shown in Figure 4. Figure 5a,b are the time-domain and frequency-domain graphs of VMD when k = 2 and α = 2000, respectively. IMF1 corresponds to the simulation signal $x_{1}$, IMF2 to the simulation signal $x_{3}$, the corresponding frequency 130 Hz of the simulation signal $x_{3}$ is not extracted, it occurs the phenomenon of under-decomposition appears. When k = 3 and α = 1.8 × 10^6^, the decomposition results of VMD are shown in Figure 6a,b. It can be seen that the signals with frequency of 130 Hz and 280 Hz are extracted, while the signals with frequency of 25 Hz are not extracted and are completely discarded. An inappropriate α will affect the accuracy of the decomposition.

**Figure 7:**
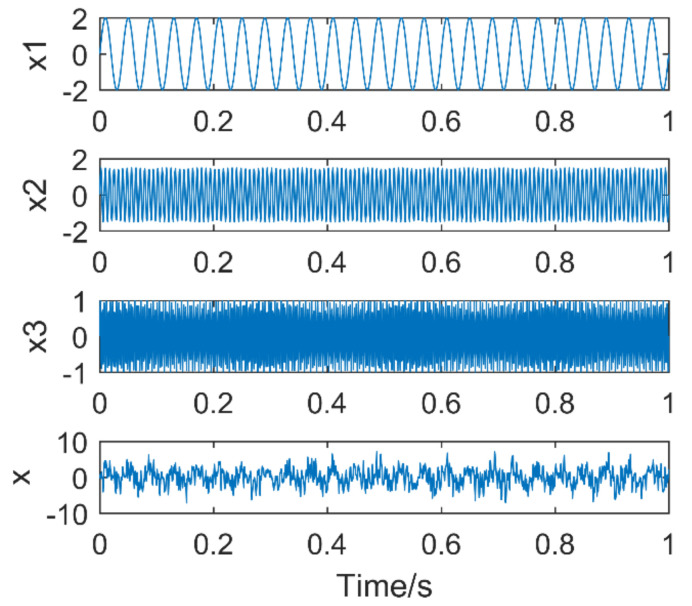
Time domain diagram of each component signal and simulation signal.

**Figure 8:**
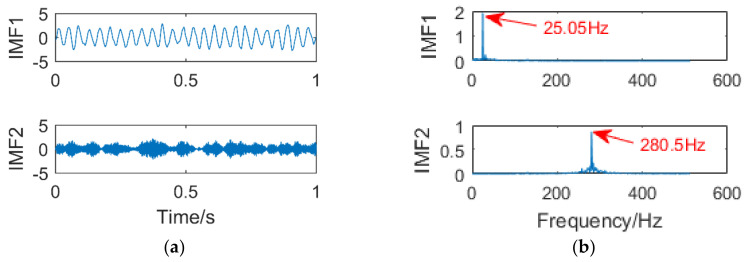
Time domain diagram (**a**) and Frequency domain diagram(**b**) of VMD

**Figure 9:**
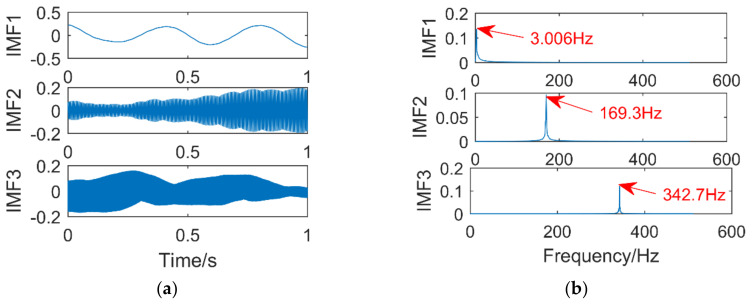
Time domain diagram (**a**) and Frequency domain diagram(**b**) of VMD

In order to explain the influence of k and α in the case of noise, random noise of amplitude 1.5 is added to formula (19), and other conditions remain unchanged. The time-domain diagram of the simulation signal is shown in Figure 7. Figure 8a,b are the time-domain and frequency-domain graphs of VMD when k = 2 and α = 2000, respectively. The result of Figure 8 is very close to Figure 6; the corresponding frequency 130 Hz of the simulation signal$x_{3}$is not extracted. Figure 9a,b are the time-domain and frequency-domain graphs of VMD, when k = 3 and α = 1.8 × 10^6^, respectively. It can be seen that a useful information is not extracted in Figure 9b.

From the analysis of the above two signals, we can draw conclusions: k and α have an effect on the decomposition of VMD, whether in a noisy or no-noise environment. Too few decomposition layers will lead to insufficient decomposition. Inappropriate α may cause inaccurate extraction of fault information or even cause misdiagnosis. 

### 3.2. Comparison of the Proposed Method with EEMD and SSD

Gearbox faults are usually in the form of modulating signals and impulse signals, and often accompanied by a lot of noise, in order to verify the feasibility of the proposed method, the following signals were constructed:

(23) $$\left\{\begin{array}{l} x_{1}\left(t\right)=2\sin\left(2\pi f_{1}t\right)\\ x_{2}\left(t\right)=1.5\times \left(1+\cos\left(2\pi f_{n}t\right)\right)\sin\left(2\pi f_{z}t\right)\\ x_{3}(t)=A_{m}\times \exp(-\frac{g}{T_{m}})\sin(2\pi f_{c}t)\\ x\left(t\right)=x_{1}\left(t\right)+x_{2}\left(t\right)+x_{3}(t)+\mathrm{noise}(t) \end{array}\right.$$

**Figure 10:**
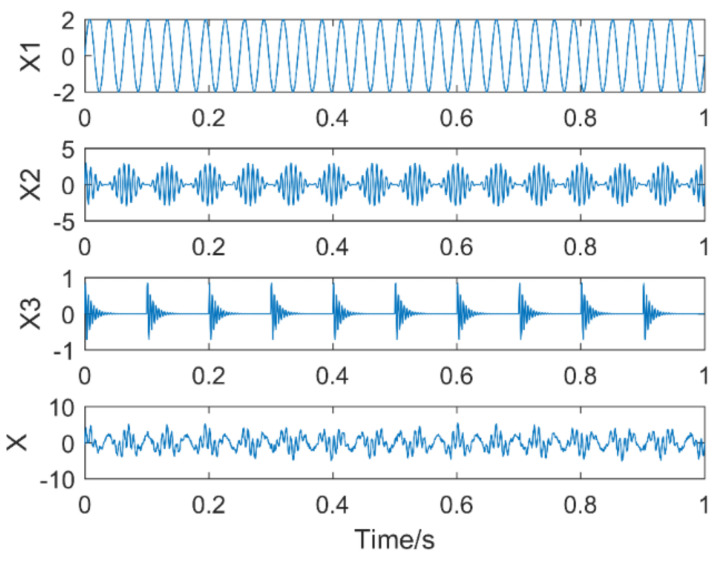
Time domain diagram of each component signal and simulation signal.

The frequency of the sinusoidal signal $f_{1}$ = 32 Hz, in the modulation signal $f_{n}$ = 15 Hz,$f_{z}$ = 130 Hz, the amplitude of the impulse signal $A_{m}$ = 1, the repetition period $T_{m}$ = 0.1, $f_{c}$ = 220 Hz, the amplitude of noise is 0.3, the number of sampling points is 1500, and the sampling frequency is 1500. Hz. The time domain plot of the analog signal is shown in Figure 10.

**Figure 11:**
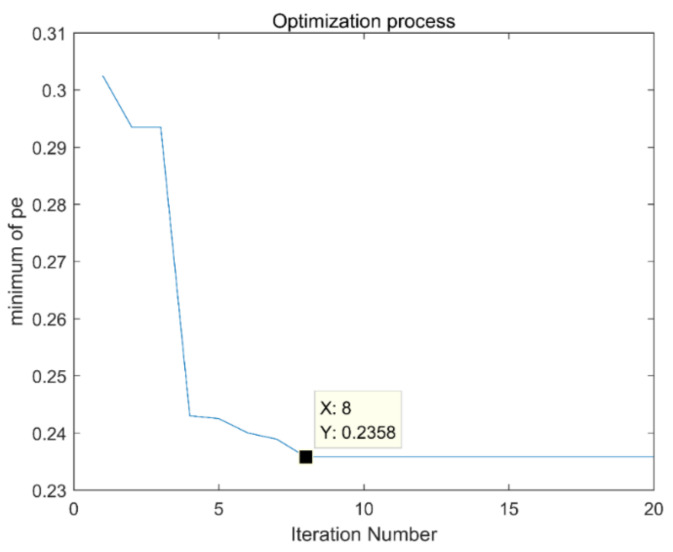
Optimization process of the immune fruit fly optimization algorithm.

**Figure 12:**
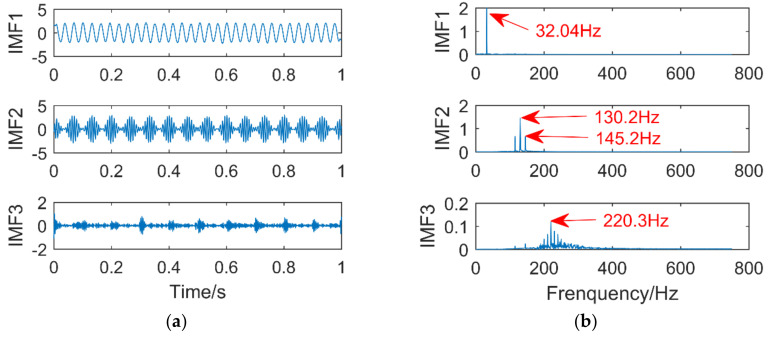
Time domain diagram (**a**) and Frequency domain diagram(**b**) of VMD.

Firstly, set the parameters of the immune fruit fly optimization algorithm: the number of fruit flies is 30, the maximum number of iterations is 20, and the number of optimal dimensions is 2. In order to reduce the amount of calculation, the optimization range is set for the parameters k and α that need to be optimized. The optimization range of k is set to [2,8], and the optimization range of α is set to [100,5000]. The dimension of permutation entropy is empirical value 6, and the optimization process is shown in Figure 11. It can be seen that the best result of optimization is in the 8th generation, and the minimum permutation entropy value of iterative optimization is 0.2358. The corresponding combination of (k, α) is [3,527], which is input into the VMD, the time domain and frequency domain diagrams obtained after decomposition are shown in Figure 12a,b.

It can be seen from Figure 12b that the IMF1 corresponds to the frequency of the sinusoidal signal of 32 Hz, IMF2 extracts the frequency of the modulated signal 130 Hz, and the sidebands of 115 Hz and 145 Hz can be clearly found in this component. Although the frequency domain diagram of IMF3 is surrounded by noise, its highest peak corresponds to the frequency of the impulse signal of 220 Hz. The error is within the allowable range, so the VMD optimized by immune fruit fly optimization algorithm can accurately extract the information of each component in the composite signal.

**Figure 13:**
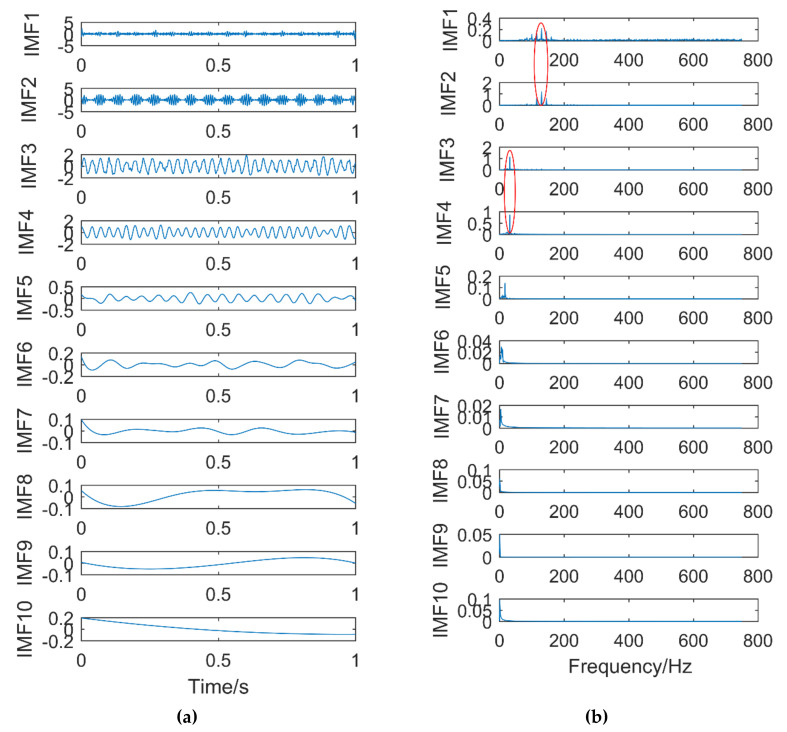
Time domain diagram (**a**) and Frequency domain diagram(**b**) of EEMD.

**Figure 14:**
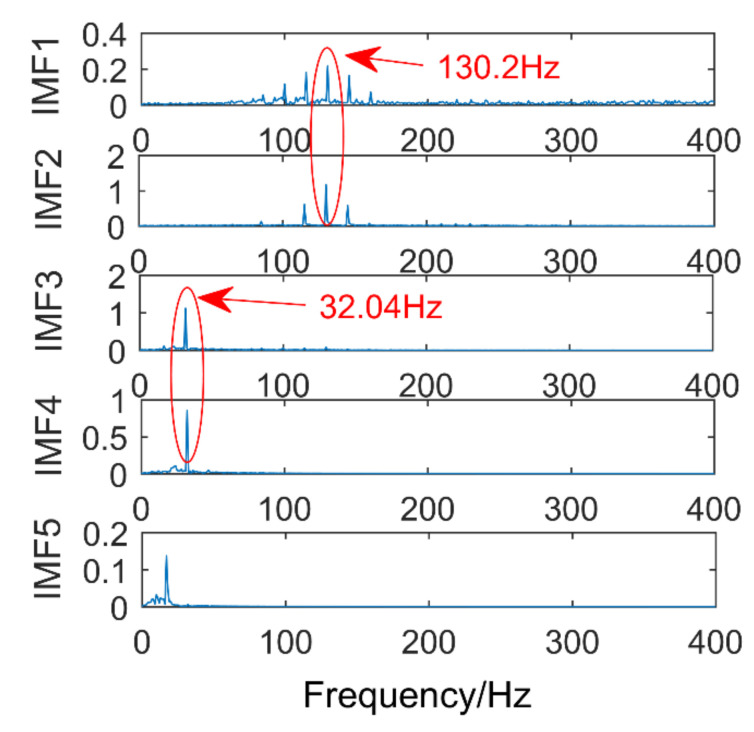
Magnified view of the first five IMFs.

Time domain and frequency domain diagrams via EEMD are shown Figure 13a,b. Figure 14 is a partial enlarged view of Figure 13b. The amplitude of gaussian white noise added by EEMD is 0.2 and the number of times added is 50. The component with frequency of 130 Hz appears in IMF1 and IMF2, it occurs mode mixing. The frequencies of IMF3 and IMF4 are both 32 Hz, mode mixing also occurred. IMF5-IMF10 are pseudo-components and fail to find signal components related to 220 Hz in all IMFs. In conclusion, severe mode aliasing occurs in EEMD, and the shock signal cannot be extracted.

**Figure 15:**
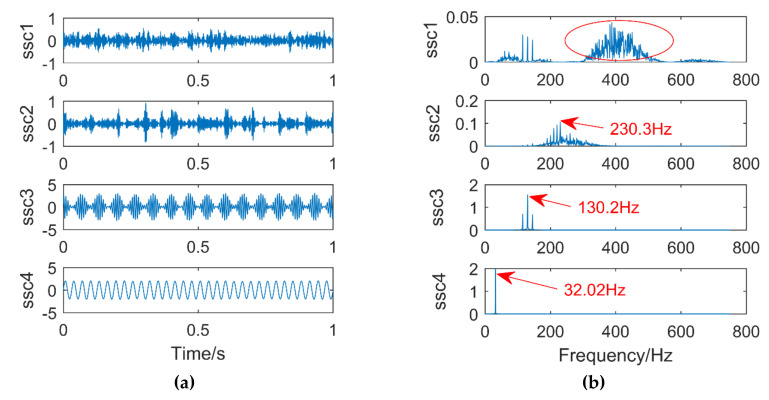
Time domain diagram (**a**) and Frequency domain diagram(**b**) of SSD.

Figure 15a,b are time domain and frequency domain diagrams after singular spectral decomposition (SSD). It can be seen from the figure that the signal is decomposed into Singular spectral components (SSC) via SSD, and high-frequency noise appears in SSC1. the frequency of the peak of SSC2 in the frequency domain diagram is 230.3 Hz, which is very different from the frequency of the impulse signal of 220 Hz, its error is 10.3 Hz, in other words, the impulse signal information cannot be accurately extracted. SSC3 extracts the modulation signal with a frequency of 130 Hz, and at the same time, the side bands with frequencies of 115 Hz and 145 Hz can be clearly seen. The sinusoidal signal with a frequency of 32 Hz appears in the SSC4. In summary, high-frequency noise occurs in the decomposition result of the SSD and the impulse signal is not accurately extracted.

## 4. Experiment

**Figure 16:**
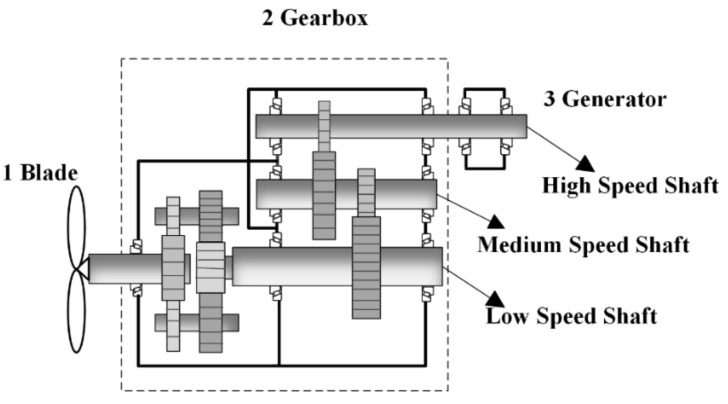
Structural diagram of gearboxs.

In order to verify the feasibility of the proposed method in engineering application, a company’s wind turbine gearbox was used for experimental verification. Its structural diagram is shown in Figure 16. It mainly includes 1 blade, 2 gearbox, and 3 generators. The gear box contains a planetary gear train, low speed shaft, medium speed shaft, high speed shaft, and so on.

**Figure 17:**
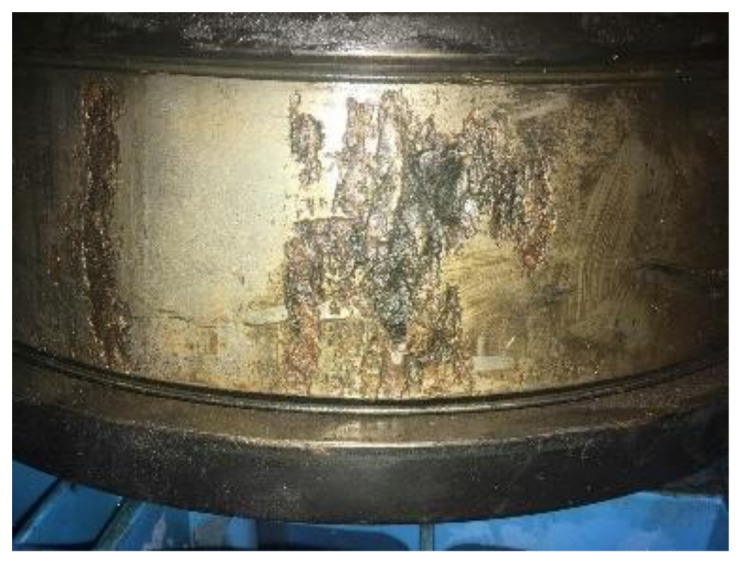
Peeling of the inner ring.

**Figure 18:**
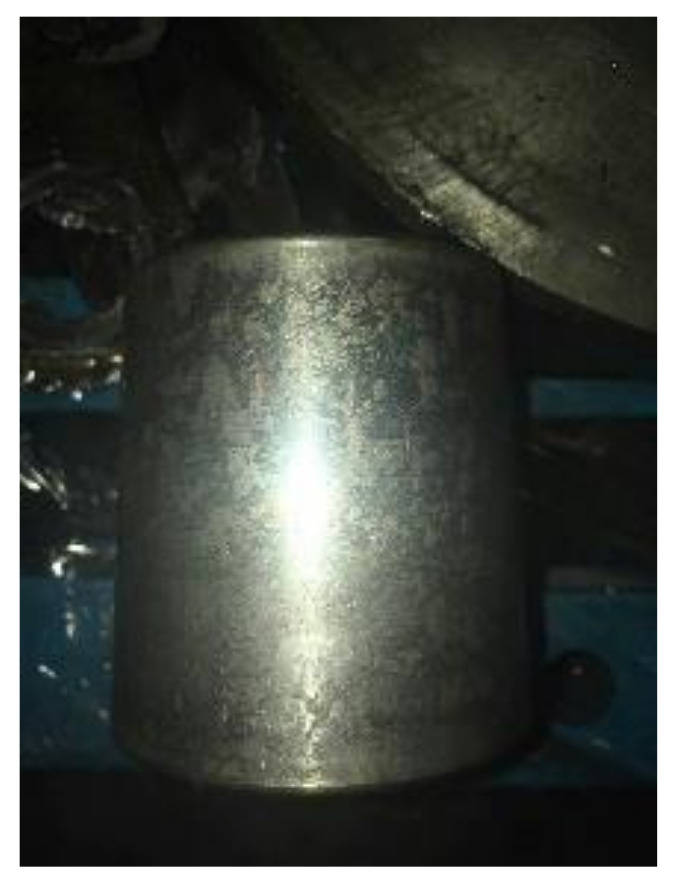
Pitting of the ball.

The faults of the gearbox occurs on the right side bearing of the intermediate speed shaft, which is the peeling of the inner ring of the bearing and the pitting of a ball. Their faults are shown in Figure 17 and Figure 18, respectively. The bearing model is 30344. The sampling frequency is 5333Hz, and the sampling point is 5333. The calculated fault frequency is shown in Table 1.

**Figure 19:**
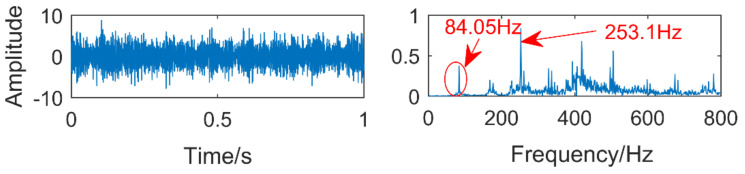
Time domain and frequency domain diagram of the vibration signal.

The time domain and frequency domain diagrams of the experimental signals are shown in Figure 19. The frequency indicated by the arrow in the frequency domain diagram is 253.1 Hz, which is very close to the triple frequency of the inner ring fault. The frequency circled by the red ellipse is 84.05 Hz, but its amplitude is lower than the corresponding amplitude of the frequency of 253.1 Hz, which is easy to cause misdiagnosis. In addition, the frequency of the ball fault cannot be found from the figure, indicating that the ball fault is weak and difficult to extract.

**Figure 20:**
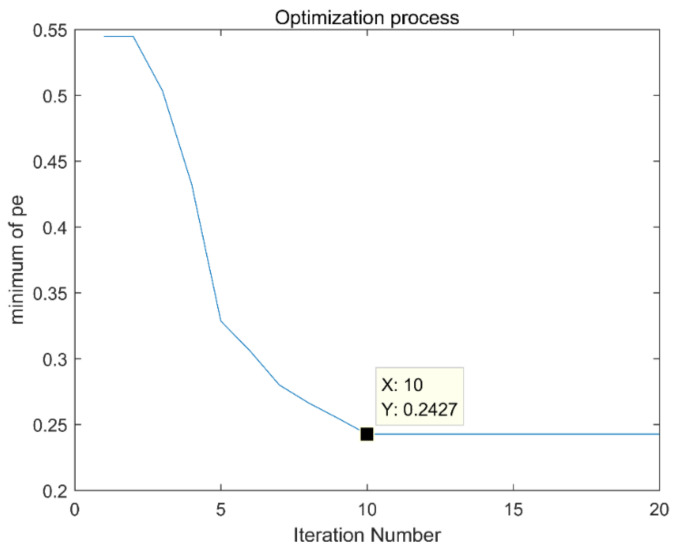
Optimization process of the immune fruit fly optimization algorithm.

**Figure 21:**
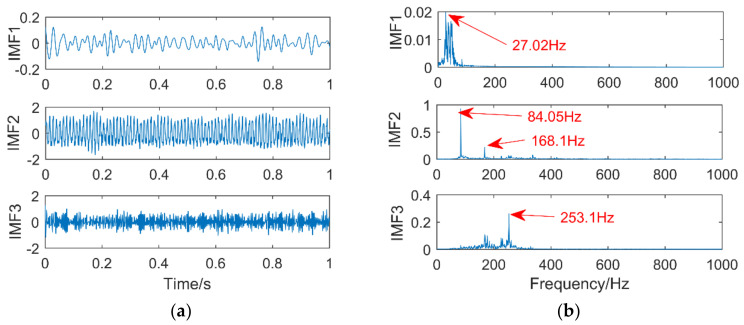
Time domain diagram (**a**) and frequency domain diagram (**b**) of VMD.

The process of optimizing the parameters of the VMD using the immune fruit fly algorithm is shown in Figure 20. The result of optimization is in the 10th generation, and the minimum permutation entropy is 0.2427. The best combination parameter for optimization is (3,657), input it into VMD, and the decomposition result is shown in Figure 21. Figure 21a shows the time domain diagram of VMD. Figure 21b shows the frequency domain diagram of VMD.

It can be seen from Figure 21b that the corresponding frequency of the IMF1 peak is 27.02 Hz. The error between it and the frequency of ball fault is 0.28 Hz, which is within the allowable error range. In the IMF2, the frequency of the inner ring fault of the bearing, and its double frequency can be clearly found. IMF3 extracts triple frequency of bearing inner ring fault. Extracted triple frequency is 0.2 Hz larger than the real triple frequency 252.9 Hz, which is very close. In summary, the VMD method optimized by immune fruit fly optimization algorithm extracts faults of the rolling body and inner ring.

## 5. Conclusions

The selection of parameters will affect the results of the VMD: Inappropriate decomposition layer k will cause over-decomposition or under-decomposition. Inappropriate α may cause the inaccurate extraction of fault information and even cause misdiagnosis. There is a certain error in using the VMD method to extract fault information. In this paper, the IFOA is applied to the VMD, and the permutation entropy value is taken as the odor concentration. The optimal combination parameters of k and α are found by the iterative method, and then the combined parameters are used for VMD and spectrum analysis is performed. The following conclusions are drawn: The pitting of the ball is weak, the frequency corresponding to the pitting of the ball cannot be found in the frequency domain diagram of the original signal, but it can be extracted by using the optimized VMD. The peeling fault of the inner ring of the bearing is strong, and the double frequency 168.1 Hz and the triple frequency 253.1 Hz can be extracted. By comparing the optimized VMD with EEMD and SSD in the simulation signal and using the measured signal in the gearbox, the feasibility of the proposed method was verified.

## Figures and Tables

**Table 1:** Fault frequency.

Rotation Speed	Rotational Frequency	Frequency of Bearing Inner Ring Fault	Frequency of the Ball Fault
491.4 rpm	8.19 Hz	84.3 Hz	27.3 Hz
